# UDP-Gal: *β*GlcNAc *β*1, 3-galactosyltransferase, polypeptide 3 (GALT3) is a tumour antigen recognised by HLA-A2-restricted cytotoxic T lymphocytes from patients with brain tumour

**DOI:** 10.1038/sj.bjc.6600593

**Published:** 2002-10-21

**Authors:** N Tsuda, Y Nonaka, S Shichijo, A Yamada, M Ito, Y Maeda, M Harada, T Kamura, K Itoh

**Affiliations:** Department of Immunology, Kurume University School of Medicine, 67 Asohi-machi, Kurume, Fukuoka 830-0011, Japan; Department of Gynecology, 67 Asohi-machi, Kurume, Fukuoka 830-0011, Japan

**Keywords:** brain tumour, immunotherapy, cytotoxic T**-**lymphocyte, tumour antigen, GALT3, cancer vaccine

## Abstract

Patient prognosis in the case of malignant brain tumours is generally poor, despite significant improvements in the early detection of the tumours, and thus the development of new treatment modalities is needed. One of the most prominent modalities is specific immunotherapy, for which the elucidation of antigenic molecules of malignant brain tumours recognized by T cells is essential. We report here a gene, UDP-Gal: βGlcNAc β1, 3-galactosyltransferase, polypeptide 3, encoding three epitope peptides recognised by tumor-reactive cytotoxic T lymphocytes in an HLA-A2-restricted manner. Two of the three peptides possessed an ability to induce HLA-A2-restricted and tumour-reactive cytotoxic T lymphocytes from peripheral blood mononuclear cells of patients with brain tumours. These peptides may be useful in the peptide-based specific immunotherapy for patients with malignant brain tumours.

*British Journal of Cancer* (2002) **87**, 1006–1012. doi:10.1038/sj.bjc.6600593 www.bjcancer.com

© 2002 Cancer Research UK

## 

Brain tumours, particularly malignant brain tumours, continue to be a major unsolved health problem in the world. Over the past decade, despite the fact that aggressive combined treatment modalities had been developed, little improvement was made in the prognosis and survival of patients with malignant glial neoplasms (Shrieve *et al*, 1999; Nwokedi *et al*, 2002). Standard management of patients with malignant glioma, especially glioblastoma multiforme, entailes surgical resection, then postoperative external beam radiotherapy (EBRT). However, because of persistent disease after both surgery and EBRT, tumour progression inevitably occurs in all patients (Fine *et al*, 1993. Therefore, the development of new therapeutic modalities for brain tumours is needed.

Recent progress in tumour immunology has clarified which molecules are involved in specific tumour immunity, including tumour rejection antigens that can be recognised by cytotoxic T-lymphocytes (CTLs) in melanomas and other cancers (van der Bruggen *et al*, 1991; Gaugler *et al*, 1994; Kawakami *et al*, 1994; Romero *et al*, 1998; Shichijo *et al*, 1998; Gomi *et al*, 1999; Yang *et al*, 1999; Kawano *et al*, 2000; Nakao *et al*, 2000; Yamada *et al*, 2001). Some of the antigenic peptides encoded by the tumour-rejection antigen genes have been used as a peptide-based vaccine in clinical trials for cancer patients, and apparent tumour regression was observed in some melanoma patients (Gaugler *et al*, 1994; Romero *et al*, 1998). Although, little information is available regarding immunotherapy in the case of brain tumours, vaccination with tumour-rejection antigens might be useful for patients with these tumours, particularly malignant gliomas.

We report here three epitope peptides derived UDP-Gal: βGlcNAc β1, 3-galactosyltransferase, polypeptide 3 (GALT3), which can be recognised by tumour-reactive CTLs in an HLA-A2-restricted manner. These peptides could be applicable in use for peptide-based therapeutic vaccine for patients with brain tumours.

## MATERIALS AND METHODS

### HLA-A2-restricted CTLs

An HLA-A2-restricted and tumour-reactive CTL line (OK-CTLs) was established from tumour-infiltrating lymphocytes of a patient with colon cancer (HLA-A0207/3101, -B46/51, -Cw1) by incubation with interleukin-2 (IL-2) (100 U ml^−1^) for more than 50 days, as reported previously (Ito *et al*, 2001). Interferon (IFN)-γ producing activity of CTLs was assessed by an enzyme-linked immunosorbent assay (ELISA, limit of sensitivity: 10 pg ml^−1^). Genotypes of HLA-class I alleles of the tumour cells have been reported (Shichijo *et al*, 1998). We investigated the surface phenotypes of CTLs using a direct immunofluorescence with fluorescein isothiocyanate (FITC)-conjugated anti-CD3 (Nu-T3), -CD4 (Nu-Th/i), or CD-8 (Nu-Ts/c) monoclonal antibodies (mAbs) (Nichirei, Tokyo, Japan). For inhibition study, we used 20 μg ml^−1^ of anti-HLA-class I (W6/32, IgG2a), anti-HLA-class II (H-DR-1, IgG2a), anti-CD8 (Nu-Ts/c, IgG), or anti-CD4 (Nu-Th/i, IgG1) mAbs.

### Tumour cell lines

Tumour cell lines used in this study were as follows: KNS60 (HLA-A2^+^ malignant glioma), KNS81 (HLA-A2^−^ malignant glioma), KALS1 (HLA-A2^+^ glioblastoma), T-98G (HLA-A2^+^ glioblastoma), no.10 (HLA-A2^+^ anaplastic astrocytoma), no.11 (HLA-A2^+^ anaplastic astrocytoma), KINGS-1 (HLA-A2^−^ anaplastic astrocytoma), SF126 (HLA-A2^+^ astrocytoma), B2-17 (HLA-A2^+^ astrocytoma), U-251 (HLA-A2^+^ astrocytoma), ONS76 (HLA-A2^+^ medulloblastoma), SW620 (HLA-A2^+^ colon adenocarcinoma), and QG-56 (HLA-A2^−^ lung squamous-cell carcinoma).

### Identification of a cDNA clone

The gene expression cloning method was used to identify genes coding tumour antigens recognised by the OK-CTLs as reported previously (Ito *et al*, 2001). A cDNA library of KNS60 glioblastoma cells was inserted into an expression vector pCMV-SPORT-2 (Invitrogen, Carlsbad, CA, USA). The cDNA of either *HLA-A0207, -A2402,* or *-A2601* was amplified by reverse transcription-polymerase chain reaction (RT–PCR) and cloned into an expression vector, pCR3 vector (Invitrogen). A full description of the transfection and screening methods was published previously (Shichijo *et al*, 1998). In brief, both 200 ng of plasmid DNA pools or clones of the cDNA library and 200 ng of *HLA-A0207* plasmid DNA were mixed in 100 μl of Opti-MEM (Invitrogen) with 1 μl of Lipofectamine (Invitrogen) and incubated for 30 min at room temperature. Subsequently, DNA mixture was transfected to COS-7 cells (5×10^3^ cells per well). Two days after cultivation, OK-CTLs (1×10^5^ cells per well) were added to the COS-7 culture. After 18 h, we collected the supernatant and measured the concentration of IFN-γ. DNA sequencing was performed with a dyedeoxynucleotide sequencing method and analysed with an ABI PRISM™ 377 DNA Sequencer (Perkin-Elmer, Foster City, CA, USA).

### Semi-quantitative analysis of GALT3 expression at the mRNA level

GALT3 expression was quantitated by RT–PCR. Total RNA was isolated from cancer cell lines (5×10^6^ cells) using RNAzol™ B (Tel-Test, Friendswood, TX, USA) according to the manufacturer's instruction. We prepared cDNA using a SuperScript™ Preamplification System for First Strand cDNA Synthesis (Invitrogen). The template cDNA was further amplified by PCR. The primers used for GALT3 amplification (nucleotides 869–1684) were as follows: sense primer, 5′-GCTGGCTTACACTGAACT-3′, and antisense primer, 5′-CGTCTTTTCTTCCCTCTCTT-3′. A primer pair used for β-actin (nucleotides 60–381) was as follows: sense primer, 5′-CTTCGCGGGCGACGATGC-3′, and antisense primer, 5′-CGTACATGGCTGGGGTGTTG-3′. PCR was performed as follows: 35 cycles for GALT3 (at 94°C for 1 min, 53°C for 2 min, and 72°C for 1 min) and 35 cycles for β-actin (at 94°C for 1 min, 58°C for 2 min, and 72°C for 1 min). The expression index of GALT3 mRNA was calculated by the following formula: index=(β-actin density of the PBMCs/β-actin density of a sample)×(GALT3 density of a sample/GALT3 density of the PBMCs).

### Peptides

We searched at the literature level for peptides capable of binding to HLA-A2 molecules (Rammensee *et al*, 1995), and 21 HLA-A2-binding peptides (purity >70%) derived from GALT3 were custom synthesised by Biologica (Nagoya, Japan) for the screening. For further studies, three peptides (GALT3_159–167_, GALT3_244–253_, GALT3_275–283_) with 95% purity were synthesised. For screening of peptides, OK-CTLs were incubated with T2 cells (an HLA-A2^+^ TAP-deficient cell line) pre-pulsed with one of the peptides (10 μM) for 18 h, and then the supernatant was collected to measure IFN-γ by ELISA.

### *In vitro* culture

We used the following method to induce CTLs. PBMCs (1×10^5^ cells per well) were incubated with 10 μM of one of the peptides in wells of 96-well micro culture plates as reported previously (Suzuki *et al*, 2002). The culture medium consisted of 45% RPMI-1640 medium, 45% AIM-V medium (Invitrogen), 10% FCS, 100 U ml^−1^ of interleukin-2 (IL-2, Shionogi, Osaka, Japan), and 0.1 μM MEM nonessential amino acid solution (Invitrogen). Half of the volume of the medium in each well was replaced every 3 days with fresh medium containing a corresponding peptide (20 μM) until day 12. The cells were harvested at day 13 and tested for their ability to produce IFN-γ in response to the corresponding peptide or control HIV peptide. The cells were further expanded in the presence of the corresponding peptide, IL-2, and irradiated autologous PBMCs as antigen-presenting cells. We examined the cells again for their surface phenotypes and measured CTL activity by a 6 h ^51^Cr-release assay at days 21–28 of the second culture.

## RESULTS

### Reactivity of the OK-CTLs to brain tumours

**Figure 1:**
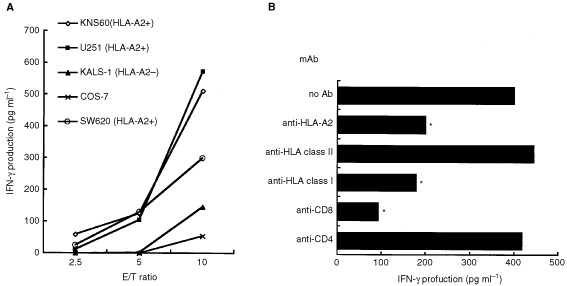
Reactivity of the OK-CTLs to brain tumours. **(A)** The OK-CTLs were tested for their ability to produce IFN-γ in response to a panel of stimulator cells at different effector to target (E/T) ratios. Target cells are KNS60 (HLA-A2^+^ malignant glioma), U251 (HLA-A2^+^ astrocytoma), SW620 (HLA-A2^+^ colon adenocarcinoma), KALS-1 (HLA-A2^−^ glioblastoma), and COS-7. **(B)** Effects of mAbs on IFN-γ production by the OK-CTLs in response to KNS60 was examined. 20 μg ml^−1^ of mAb was added to the culture. Values represent the mean of the triplicate determinants. *P<0.05.

Reactivity of the OK-CTLs, which was established from TILs of colon cancer patient to brain tumour cell lines, was examined to know whether this CTL lines is suitable in use for identification of CTL-directed antigens. The detailed characteristics of the OK-CTLs were previously reported (Ito *et al*, 2001). The OK-CTLs produced significant amounts of IFN-γ in response to *HLA-A2*^+^ brain tumour cells, including KNS60 (malignant glioma) and U-251 (astrocytoma), as well as colon cancer cells (SW620), but not to HLA-A2^−^ KALS-1 (glioblastoma) and COS-7 cells (Figure 1A

). IFN-γ production was inhibited by the addition of anti-HLA-class I (W6/32), anti-CD8, or anti-HLA-A2 mAb, but not by that of anti-HLA-class II or anti-CD4 mAbs (Figure 1B). These results suggest that the OK-CTLs specifically recognised brain tumour cells in an HLA-A2-restricted manner through an interaction between class-I and CD8 molecules, and these were used as the effector CTLs in the following experiments.

### Cloning of a cDNA encoding a tumour antigen

**Figure 2:**
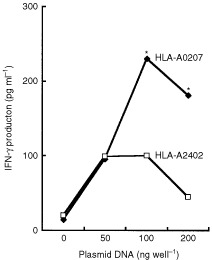
Recognition of a product encoded by the cDNA clone by the OK-CTLs. Indicated amounts of the cloned cDNA and 100 ng of *HLA-A0207* or *-A2402* cDNA were co-transfected to COS-7 cells, followed by a test of their ability to stimulate IFN-γ production by the OK-CTLs. The background of IFN-γ production by the CTLs in response to COS-7 cells (under 100 pg ml^−1^) was subtracted. *P<0.05.

**Figure 3:**
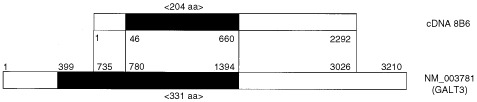
Comparison of sequences of cDNA 8B6 and *GALT3*. The black box represents the coding region.

To identify genes encoding KNS60-derived antigens recognised by the OK-CTLs, a total of 1×10^5^ cDNA clones from the cDNA library of KNS60 was screened. The cDNA library was co-transfected with *HLA-A0207* cDNA to COS-7 cells followed by a test of their ability to stimulate IFN-γ production by the OK-CTLs. After repeated screenings, one cDNA clone 8B6 (2292 bp) was identified. Namely, the cDNA clone stimulated the OK-CTLs in a dose-dependent manner when the cDNA was co-transfected with *HLA-A0207* but not when it was co-transfected with control *HLA-A2402* (Figure 2

). The nucleotide sequence of the clone 8B6 (GeneBank accession number is AB060691) was identical to the partial sequence of the UDP-Gal: β*GlcNAc* β*1, 3-galactsyltransferase, polypeptide 3 (GALT3)* gene (GeneBank accession number is NM_003781) (Figure 3

). The cDNA 8B6 encoded 204 amino acids, which were identical to the C-terminal amino acids of GALT3. These results indicate that the *GALT3* gene encoded an antigen that was recognised by the OK-CTLs.

### mRNA expression of *GALT3*

**Figure 4:**
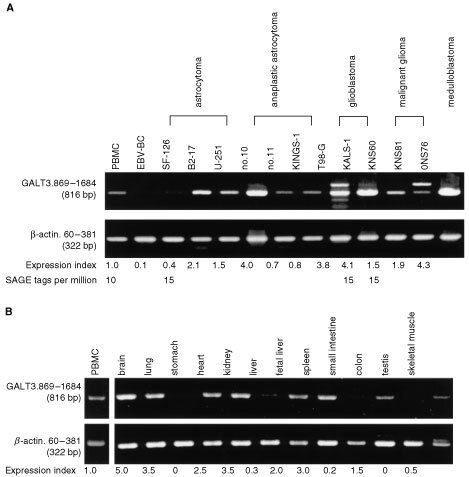
**(A)** Semi-quantitative RT–PCR analysis of the expression of GALT3 in brain tumour cell lines. Calculation method of the expression index is shown in the Materials and Methods. SAGE tags per million are cited from SAGE database. **(B)** Expression of GALT3 mRNA in the normal tissues.

The expression of *GALT3* in normal tissues and tumour cell lines was analysed by semi-quantitative RT–PCR. *GALT3* mRNA was found in all of the brain tumour cell lines tested (three astrocytomas, three anaplastic astrocytomas, two glioblastomas, two malignant gliomas, and one medulloblastoma) (Figure 4A

). The expression of *GALT3* in these cell lines varied, and no correlation with histological types of original tumours was observed. In contrast, it was not expressed in Epstein-Barr-Virus-transformed B cell line (EBV-BC). The highest expression was observed in normal brain tissue (Figure 4B). Relatively strong expression of *GALT3* was also observed in several normal tissues, such as lung, kidney, and spleen, whereas it was not expressed at all in stomach and testis. The expression of *GALT3* in several SAGE (serial analysis of gene expression) libraries of brain tumours and other cancers including colon cancer has been reported in a database of the National Center for Biotechnology Information, NIH (data not shown). The GALT3 expression in the normal brain was also confirmed by SAGE database analysis.

### Identification of immunogenic epitopes of GALT3 capable of inducing CTLs

**Figure 5:**
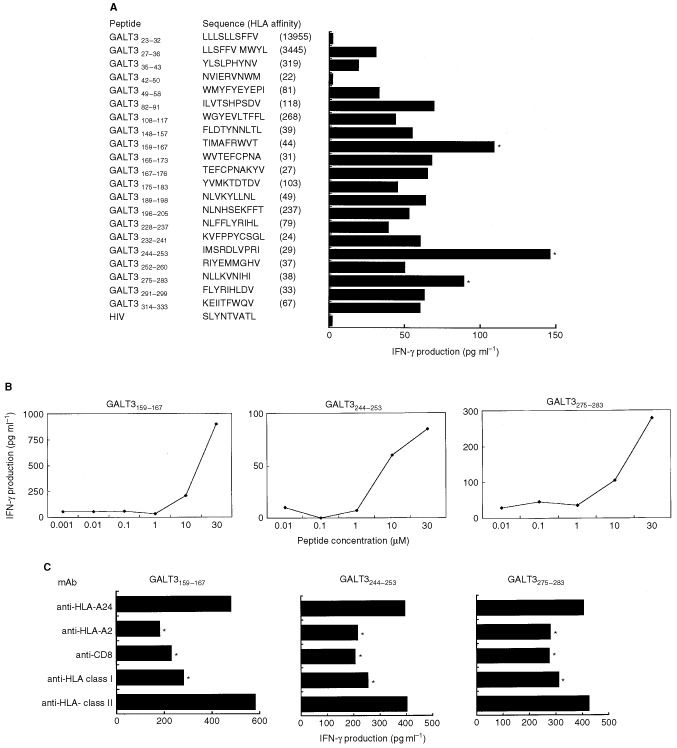
**(A)** Identification of the GALT3-derived peptides recognised by the OK-CTLs. Each of the 21 peptides derived from GALT3 was loaded onto T2 cells at a concentration of 10 μM for 2 h. The OK-CTLs were then added at an E/T ratio of 10, incubated for 18 h followed by collection of the supernatant for measurement of IFN-γ. Values indicate the mean of triplicate assay. The background (under 100 pg ml^−1^) of IFN-γ production by the OK-CTLs in response to the unloaded T2 cells was subtracted from the values in this figure. The ‘HLA affinity’ in the parenthesis of the figure indicates HLA-A2 binding affinity of each peptide on the database. **(B)** Dose-dependency of the GALT3-derived peptides on IFN-γ production by the OK-CTLs. Indicated doses of the GALT3 peptides were loaded onto T2 cells for 2 h followed by a test of their ability to stimulate IFN-γ production by OK-CTLs. Values indicate the means of the triplicate assays. **(C)** Effects of mAb on the peptide recognition by the OK-CTLs. Values indicate the means of the triplicate assays. *P<0.05.

Twenty-one GALT3-derived peptides, possessing HLA-A2-binding motifs, was loaded on T2 cells, and its ability to induce IFN-γ production by the OK-CTLs was examined. Although the isolated cDNA 8B6 does not contain the sequence encoding the N-terminal amino acids of GALT3, peptides from whole GALT3 were included in the peptide screening. Three peptides, GALT3_159–167_ [TIMAFRWVT], GALT3_244–253_ [IMSRDLVPRI], and GALT3_275–283_ [NLLKVNIHI], induced significant levels of IFN-γ production by the OK-CTLs (Figure 5A

). The stimulating effects of these peptides was dose-dependent, and the effect was observed in more than 10 μM of concentration for peptide loading on T2 cells (Figure 5B). It was further tried to confirm that GALT3-reactive T cells were CD8^+^ and HLA-A2-restricted. An addition of anti-HLA-A2, anti-HLA-class I, or anti-CD8 mAb significantly suppressed the response, but no suppression was observed by the addition of anti-HLA-class II (HLA-DR) mAb (Figure 5C). These results suggested that the OK-CTLs recognised the GALT3-derived peptides in association with HLA-A2 molecules.

### CTL induction by the GALT3-derived peptides from patients with brain tumours

**Figure 6:**
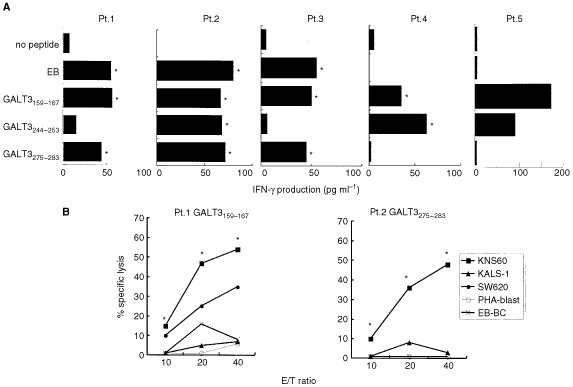
Induction of brain tumour-reactive CTLs by the GALT3-derived peptides. **(A)** PBMCs from four brain tumour patients (Pt.1,2. glioblastoma, Pt.3. metastatic brain tumor, Pt.4. meningioma) and one lung cancer patient (Pt.5) were stimulated with one of the three GALT3-derived peptides (10 μM) and IFN-γ production in response to the corresponding peptide was examined. **(B)** PBMCs of two brain tumour patients, which were stimulated with either the GALT3_159–167_ or GALT3_275–283_, were examined for their cytotoxicity against four target cells. Values represent the means of triplicate assay. *P<0.05.

We tested these three GALT3 peptides to determine their ability to induce CTLs from six patients with brain tumours (four glioblastomas, one metastatic brain tumour, and one meningioma) and six healthy donors. Their PBMCs were stimulated six times *in vitro* with one of the three peptides (GALT3_159–167_, GALT3_244–253_, and GALT3_275–283_), and IFN-γ production in response to the corresponding peptide was analysed (Figure 6

). EBV-derived peptide (GLCTLVAML) capable of binding to HLA-A2 molecules was used as a positive control as reported previously (Suzuki *et al*, 2002). The PBMC culture of five patients (two glioblastomas, one metastatic brain tumour, one meningioma, and one lung cancer) produced significant levels of IFN-γ in response to the corresponding peptide-loaded T2 cells (Figure 6A). In contrast, none of the PBMC cultures of six healthy donors produced IFN-γ in response to the corresponding peptide stimulation except for the EBV peptide (data not shown). Cytotoxic activity of the peptide-reactive T cells was further examined by a 6 h ^51^Cr-release assay, and representative results are shown in Figure 6B. The peptide-reactive T cells from PBMCs of two patients stimulated with either GALT3_159–167_ or GALT3_275–283_ showed significant levels of cytotoxicity against HLA-A2^+^ KNS-60 and SW620 (colon cancer), but not HLA-A2^−^ KALS-1, HLA-A2^+^ EBV-B cells, or autologous PHA-blasts. In contrast, no cytotoxic activity was observed in GALT3_244–253_ induced PBMCs (data not shown).

## DISCUSSION

We identified that GALT3 was an antigen recognised by HLA-A2-restricted and brain tumour-reactive CTLs. GALT3 was expressed at mRNA levels in all of the brain tumour cell lines tested. The message was also detectable in several normal tissues with the highest expression in brain. The GALT3, also termed β3GalNAc-T1, possessed a globoside (Gb4) synthase activity (Amado *et al*, 1998; Okajima *et al*, 2000). Globoside is the most prominent neutral glycosphingolipid in both cerebromicrovascular endothelial cells and erythrocytes. Brain tumours, such as pilocytic astrocytomas and pleomorphic xanthoastrocytomas, also contain a high proportion (>15%) of globoside in total neutral glycolipids (Yates *et al*, 1999). A function of globoside as an initiator of signal transduction associated with cell adhesion has been reported (Song *et al*, 1998). Interestingly, globoside has also been reported as a tumour-associated glycosphingolipid antigen defined by a monoclonal antibody (Hakomori, 1998). Namely, clustered glycosphingolipid antigens organised with transducer molecules in the microdomain have been found to comprise a structural and functional unit involved in tumour cell adhesion coupled with signal transduction, and they may also initiate the metastatic process.

We isolated a cDNA clone which is partially identical to the *GALT3* gene. In addition, several bands were observed when RT–PCR was done on two brain tumour cell lines, as shown in Figure 4A. These results may indicate that the *GALT3* gene could be alternatively spliced, especially in tumour cells, or have a gene family. Further study is needed to elucidate this observation.

We identified three GALT3-derived peptides that can be recognised by HLA-A2-restricted CTLs, and two of them possessed an ability to induce HLA-A2-restricted and tumour-reactive CTLs in PBMC culture of patients with brain tumours, but not in PBMC culture of healthy donors. These CTLs did not show cytotoxicity to either HLA-A2^+^ normal lymphoid cells (PHA-blast) or EBV-B cells. Several studies suggest that, at least in some circumstances, glioma cells can be recognised by CTLs or other immunocompetent cells *in vivo,* despite the location of glioma cells in the central nervous system (CNS), which is believed to be an immunologically-privileged site (Yates, 1986; Hickey *et al*, 1991). It has also been reported that brain-tumour vessels lose their blood-brain barrier features after interaction with immune cells (Owens *et al*, 1994), and activated T cells cross the brain–blood barrier of such vessels (Hafler and Weiner, 1987). In addition, significant trafficking of activated T cells throughout the CNS has been reported (Yates, 1986; Hickey *et al*, 1991). Those results together with the results in the present study suggest that peptide-based immunotherapy is feasible in patients with brain tumours. The GALT3 peptides may also be applicable for other cancers, since peptide-specific CTLs could be induced in PBMCs of a lung cancer patient (Figure 6A) and the GALT3 expression was observed in various tumour cell lines (data not shown). Vaccination of GALT3 peptide for patients with brain tumour might induce adverse events because of its expression in normal brain and the other normal tissues. However, it should be noted that no severe adverse effects in normal tissues or organs have been reported in the clinical trials of cancer vaccines specific to the MAGE-1, MAGE-3, Melan-A, gp100, tyrosinase, and NY-ESO-1 in melanoma patients, although these molecules are expressed in the normal testis, retina, and/or melanocytes at both the mRNA and protein levels (Hu *et al*, 1996; Marchand *et al*, 1998; Rodolfo and Colombo, 1999; Thurner *et al*, 1999; Mackensen *et al*, 2000; Jager *et al*, 2001). Similarly, no severe adverse effects on the function of normal organs have been observed in our clinical trials of peptide-based cancer vaccines for colon cancer patients, even though some of the target molecules are ubiquitously expressed in normal colon (Miyagi *et al*, 2001). Intracellular traffic of antigenic molecules and subsequent processing of the antigenic peptides in proteasomes of normal cells may differ from that of tumour cells in these cases. Alternatively, some molecules in normal cells, including a family of serpins (a group of serine-protease inhibitors), might be involved in resistance of normal cell to CTL-mediated lysis.

In conclusion, we identified that GALT3 could be recognised by HLA-A2-restricted and tumour-reactive CTLs and that two GALT3 peptides were capable of inducing brain tumour-reactive CTLs from HLA-A2^+^ patients with brain tumours. In general, the HLA-A2 allele is found in 53% of Chinese, 40% of Japanese, 49% of Northern America Caucasians, 38% of Southern America Caucasins, and 23% of African Blacks (Imanishi *et al*, 1992). These results suggest that these GALT3-derived peptides could be a candidate for therapeutic vaccines for a relatively large number of brain tumour patients with HLA-A2 molecules in the world.
